# CuS-PNIPAm nanoparticles with the ability to initiatively capture bacteria for photothermal treatment of infected skin

**DOI:** 10.1093/rb/rbac026

**Published:** 2022-04-29

**Authors:** Zizhen Wang, Zishuo Hou, Peiwen Wang, Fan Chen, Xianglin Luo

**Affiliations:** 1 College of Polymer Science and Engineering, Sichuan University, Chengdu, People’s Republic of China; 2 State Key Laboratory of Polymer Materials Engineering, Sichuan University, Chengdu, People’s Republic of China

**Keywords:** capturing bacteria, wound healing acceleration, photothermal ablation, ion-controlled release

## Abstract

Copper sulfide nanoparticles (CuS NPs) have shown great potential in various application fields, especially in biomedical engineering fields. CuS NPs, with the ability to actively capture and kill bacteria and without the worry of biocompatibility, will greatly expand their applications. Herein, a four-arm star thermo-sensitive polyisopropylacrylamide (4sPNIPAm) was used to modify CuS NPs (CuS-PNIPAm NPs). The obtained NPs displayed the controlled release of copper ions and higher photothermal conversion ability in comparison with contrast materials CuS-PEG NPs and CuS NPs. Aggregation of CuS-PNIPAm NPs at above 34°C resulted in capturing bacteria by forming the aggregates of NPs-bacteria. Both *Staphylococcus aureus* and *Escherichia coli* co-cultured with CuS-PNIPAm NPs were completely killed upon near-infrared irradiation in minutes. Furthermore, CuS-PNIPAm NPs were verified to be a photothermal agent without toxic effect. In *in vivo* experiment, the NPs effectively killed the bacteria in the wound and accelerated the process of wound repairment. Overall, photothermal treatment by CuS-PNIPAm NPs demonstrates the ability to actively capture and kill bacteria, and has a potential in the treatment of infected skin and the regeneration of skin tissues. The therapy will exert a far-reaching impact on the regeneration of stubborn chronic wounds.

## Graphical Abstract

**Figure:**
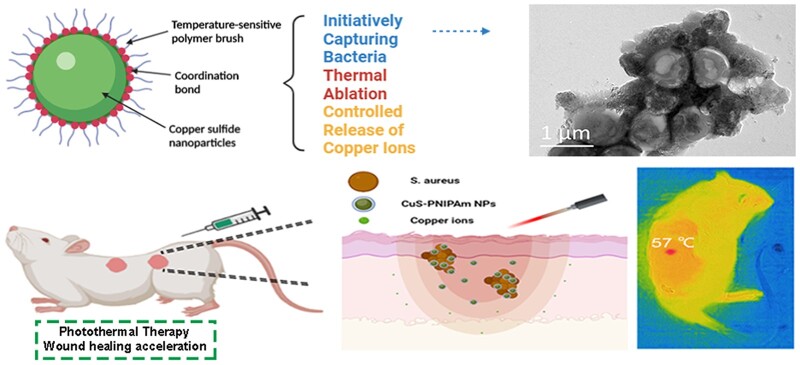

## Introduction

Copper sulfide nanoparticles (CuS NPs) have excellent photoelectric properties and lower cost compared with other metal nanomaterials [1], thus have been widely used in charge transmission, heat conduction, photocatalysis and light emission [2, 3]. In biomedical engineering fields, CuS NPs have recently received a lot of attention because of that they have a wide absorption in 700–1100 nm near-infrared (NIR) region. The strong absorption peak of CuS NPs can be expediently adjusted to above 900 nm by changing only the concentration [4]. Thus, CuS NPs can be applied in photothermal therapy (PTT) and have high photothermal efficacy [5]. For example, Wang *et al*. [6] used surface-functionalized modified CuS NPs both as photothermal mediators for tumor hyperthermia and as an absorbent of tumor antigens to induce antitumor immune response. In recent years, PTT, used to treat bacterial infections, has attracted great interest of researchers, since antibacterial therapy conducted by PTT does not lead to the formation of drug-resistant bacterial pathogens [7–11]. As a good photothermal agent, CuS NPs have made burgeoning advances in antibacterial treatment. CuS nanodots not only synergistically treated drug-resistant bacterial infections, but also accelerated wound healing via remote control of copper-ion release by photothermal effect [12]. Impressively, CuS NPs modified by a polymer with quaternary ammonium groups achieved effective capture and photothermal ablation of bacteria by actively adsorbing bacteria [13]. In addition, when CuS NPs were added into hydrogels, the synergistic antibacterial effect and skin regeneration under wet environment were attained through sterilization of copper ions and PTT [14, 15]. Therefore, CuS NPs have great application prospects in antibacterial field and tissue regeneration aspect of infected skin.

When CuS NPs are utilized for biomedical applications, it is very important to slow the spread of released copper ions in order to control the ion concentration within the acceptable range of human body. The concentration of copper ions in cells generally is around 10^−5^–10^−4^ M [16], and toxic side effects occur when the concentration is higher than that. Furthermore, copper ions may activate the carcinogenic signaling pathway and promote the occurrence of tumors [17]. As a metal sulfide, the stability of CuS NPs is poor, especially in light, and is not conducive to storage and practical applications. In addition, CuS NPs generally do not possess the ability to initiatively capture bacteria. Although the strategy via surface modification of CuS NPs by a polymer of quaternary ammonium can realize active adsorption of bacteria [13], the existence of abundant positive charges on the NP surface may lead to the apoptosis and hemolysis of normal cells [18]. Studies have shown that quaternary ammonium salt in water can affect the normal development of animal embryos [19–21]. Undoubtedly, to stabilize CuS NPs and endow them with the ability to initiatively capture bacteria by using a biocompatible polymer will greatly expand the applications of CuS NPs in the field of tissue regeneration in infected wounds.

Polyisopropylacrylamide (PNIPAm) is a neutral temperature-sensitive biocompatible polymer, which has been widely used in intelligent responsive surface, drug carrier and functional hydrogels [22–24]. PNIPAm can be applied for the avoidance of bacterial adhesion and adjustment of cell adhesion on hydrogels via turning the temperature down or up than its minimum critical co-dissolution temperature (LCST) [25, 26]. For nanometer materials, Fe_3_O_4_-carbon nanotubes with PNIPAm brushes on the surfaces realized the goals of effectively killing bacteria by PTT and removal of dead bacteria by magnetic response [27]. Considering the existence of hydrophobic C-C main chains and hydrophilic amides in PNIPAm [27], we speculated that PNIPAm would well encapsulate CuS NPs, reduce the release of copper ions, and simultaneously display the character of active bacteria capturing above its LCST.

Here, in order to modify CuS NPs to form stable copper sulfide NPs (CuS-PNIPAm NPs), a four-arm star-shaped thermal polymer (4sPNIPAm) was designed, with the consideration of that 4sPNIPAm not only owns the performances of PNIPAm, but also possesses stereoscopic effect of a four-arm polymer while maintaining the interaction between metal ions and disulfide of 4sPNIPAm. After the synthesis of CuS NPs and 4sPNIPAm, studies on the photothermal properties, stability of NPs, the release of copper ions, antimicrobial activity, mechanism against Gram-negative and Gram-positive bacteria, and biocompatibility of CuS-PNIPAm NPs were proceeded. This work illustrates a typical example of using 4sPNIPAm to quick modify metal sulfide NPs to enhance the stability of NPs, and also highlights the potential of using CuS-PNIPAm NPs as a novel antimicrobial agent with actively capturing function. The results show that CuS-PNIPAm NPs not only demonstrate good photothermal treatment effect to infected skin, but also accelerate wound regeneration.

## Experimental

### Materials

CuCl_2_·2H_2_O, other common chemicals and solvents were analytical grade and obtained from KeLong Chemical Co., Ltd (Chengdu, China). N-isopropylacrylamide (with 200 ppm MeHQ), tris[2-(dimethylamino) ethyl] amine (Me_6_TREN > 98.0%), 2-bromo-2-methylpropionyl bromide (97%) were purchased from Aladdin (Shanghai, China). Agar, tryptone, copper (I) bromide (CuBr, 99%) and 3- (4,5-dimethylthia-zol-2-yl)-2,5-diphenylte-trazoliumbromide (MTT) were purchased from Sigma. Thiol poly-ethylene glycol amino (SH-PEG2000-NH_2_) was bought from Shanghai Toyongbiotech. Inc. Phosphate-buffered saline (PBS), penicillin, strephomycin, Dulbecco’s modified eagle medium (DMEM), tryisin and fetal bovine serum were purchased from Hyclone. Mouse Fibroblasts L929, *Escherichia coli* and *Staphylococcus aureus* were obtained from the West China Hospital.

### Synthesis and characterization of 4sPNIPAm

Poly (N-isopropylacrylamide) with four arms (4sPNIPAm) was synthesized by atom transfer radical polymerization (ATRP) of NIPAm. First, the initiator (4s-Br) was synthesized as previously reported [28–30], and the appropriate improvements were made as shown in Supplementary Fig. S1. Then, 4s-Br, NIPAm and Me6TREN were dissolved in a 12 ml mixture of DMF/H_2_O (3/1, V/V), and the solution was bubbled with nitrogen for 30 min. CuBr was added into the reactor, and the reaction was conducted under a nitrogen atmosphere at room temperature for 24 h. Then, the reaction mixture was purified by dialysis for 2 days, and the product was obtained by freeze-dying. The synthesized polymers were characterized by nuclear magnetic resonance (NMR, Unity Inova 400 spectrometer), attenuated total reflection Fourier transform infrared spectroscopy (ATR-FTIR, Magna 560 FTIR spectrometer), and gel permeation chromatography (HLC-8320GPC, TOSON).

### Preparation and characterization of CuS-PNIPAm NPs

The NPs were synthesized according to previously described method [6]. In brief, 17.8 mg of CuCl_2_·2H_2_O and 22.8 mg of sodium citrate dihydrate were dissolved in 100 ml of deionized water. Then 24.0 mg of Na_2_S·9H_2_O was added. The mixture was stirred at 90°C for 2 h under N_2_ atmosphere. When the color of the solution turned to dark-green, CuS NPs (Cit-CuS NPs) completely formed. 4sPNIPAm and NH_2_-PEG5000-SH (0.1 mM) were added into the above mixture under stirring overnight at 25°C to CuS NPs coated by the polymers. CuS NPs, CuS-PEG NPs and CuS-PNIPAm NPs were purified through the dialysis method (MWCO 35000). The obtained CuS NPs, CuS-PEG NPs and CuS-PNIPAm NPs were characterized through dynamic light scattering (DLS), transmission electron microscopy (TEM), thermal gravimetric analyzer (TGA, Netzsch), X-ray diffraction (XRD, Ultima IV, Rigaku) and X-ray photoelectron spectroscopy (XPS, XSAM800, Kratos Analytical).

### Photothermal property of CuS-PNIPAm NPs

An UV/visible spectrophotometer (Specord 200 plus, Analytik Jena) was used to characterize the 400–900 nm absorbance of CuS NPs, CuS-PEG NPs and CuS-PNIPAm NPs with CuS concentrations of 0.5–0.1 mM.

The infrared thermal images and temperature changes of CuS NPs, CuS-PEG NPs and CuS-PNIPAm NPs were recorded with an infrared thermal imaging camera (FLIR ONE, FLIR Systems, Inc., USA) under laser irradiation (808 nm, 2 W cm^−2^, 100 s). In detail, 200 μl of the NPs were severally added into 1.5 ml pointed PE tubes, and the laser was placed at 20 cm right above the tube. Furthermore, the photothermal properties of CuS-PNIPAm NPs with three cycles of laser on and off and 1- to 10-fold dilution were measured.

### Bacteria capture performances

The photothermal antibacterial efficiencies of the NPs were investigated by using *E.coli* and *S.aureus* as model bacteria. The NPs solutions (100 μl, 0.16 mM) were respectively dispersed in 10^7^ colony forming unit (CFU) bacteria suspensions (100 μl), and then irradiated with 808 nm NIR at a power density of 2 W/cm^2^ for 5 min. Then, the bacterial suspension of different groups was centrifuged to collect the bacteria. The bacteria were fixed with 4% paraformaldehyde for 2 h, and then sequentially dehydrated with ethanol (30%, 50%, 70%, 90% and 100%). Then, the morphologies and EDAX mapping were observed by SEM. To prepare TEM samples, NP solutions with bacteria before and after NIR irradiation are dropped on copper grid, slightly dried at either 25°C or 55°C to maintain the morphologies of the NPs–bacteria aggregations.

### *In vitro* antibacterial test

*Staphylococcus* *aureus* and *E.coli* (100 μl, 1 × 10^7^ CFU/ml) in logarithmic growth phase were added separately into 1.5 ml pointed PE tubes. The NPs solutions (100 μl, 0.16 mM) were respectively dispersed in the tubes and then irradiated with 808 nm NIR at a power density of 2 W/cm^2^ for 5 min. Subsequently, the bacterial suspensions from the wells were diluted by 20 times, and the diluted suspensions were put on the Luria–Bertani (LB) ager culture dishes and cultured for 24 h at 37°C. Then, the colony units on LB ager dishes were recorded by a camera.

Then, the suspension diluted with LB was incubated at 37°C for 12 h, and the absorbance was measured by UV-vis spectroscopy at 600 nm per 2 h to study the growth of the bacteria.

### Release of copper ions *in vitro*

Two milliliter of CuS NPs, CuS-PEG NPs and CuS-PNIPAm NPs were stored in dialysis bags and immerged to 20 ml PBS at 37°C. At specific time points, 2 ml release medium was collected and replaced by 2 ml fresh PBS. The concentration of Cu^2+^ was measured by an inductively coupled plasma-atomic emission spectrometer.

### Biocompatibility test of NP solutions

After L929 were cultured in 96-well plates at 37°C in 5% CO_2_ atmosphere for 24 h, the medium in each well was replaced by 100 μl of the NP solutions. The cells co-cultured with fresh DMEM were set as a control group (*n* = 5). The cells were cultured for another 24 h, then MTT assay was used to measure the cell viability. Cell viability = *A_t_*/*A*_0_ × 100%, where *A_t_* is the absorbance of experimental groups at 492 nm, *A*_0_ represents the absorbance of the control group.

Hemolysis assay was performed to evaluate the blood compatibility of the NPs. The arterial blood of healthy female Sprague Dawley (SD) rats was collected, and the Red Blood Cells (RBCs) were obtained by refrigerated centrifugation. 800 μl of the diluted RBCs (10% RBCs precipitate in PBS) was added to a centrifuge tube with 100 μl of the NPs solutions and incubated at 37°C for 1 h. Normal saline and distilled water added to RBCs were used as a negative control and a positive control, respectively. After 5 min centrifugation at 3500 rpm, photos of the tubes were taken, and incidentally, the RBCs were photographed by an IX71 microscope with a magnification of 40 times. The supernatant of each sample was tested at 540 nm (*n* = 3). Hemolysis ratio= (*As*–*An*)/(*Ap*–*An*) × 100%, where *As* is the absorbance of experimental groups, *An* is the absorbance of the negative control group and *Ap* is the absorbance of the positive control group.

### Wound healing assay

SD rats (200–250 g) were obtained from Chengdu Dashuo Biological Technology Co., Ltd. All the experiments were performed by following the Guidelines for Care and Use of Laboratory Animals of Sichuan University. Four full thickness-round wounds with 10 mm diameter at the back of each SD rat were created by a sharp round pouch (*n* = 5). Then 100 μl *S.aureus* suspension (1 × 10^8^ CFU/ml) was added to each wound. The infected wounds were treated PBS, CuS NPs, CuS-PEG NPs, CuS-PNIPAm NPs (200 μl, 0.2 mM) with 808 nm laser for 5 min, respectively. The wound images were recorded by a camera at 0, 2, 4, 6, 8 and 10 days, and the wound area were measured by Image J. In the end, all of the rats were sacrificed, and the tissues around the wounds were excised. The tissue sections were fixed with 10% formalin and stained with H&E staining. Skin tissue regeneration was investigated by histologic evaluation.

### Statistical analysis

The experiment data were demonstrated by mean ± standard deviation. The data analysis was statistically realized by GraphPad Prism. Difference analysis was used by Student’s t-test. The data were significantly different when *P < 0.05, **P < 0.01 and ***P < 0.001.

## Results and discussion

### Synthesis and characterization of 4SPNIPAm

**Figure 1.**
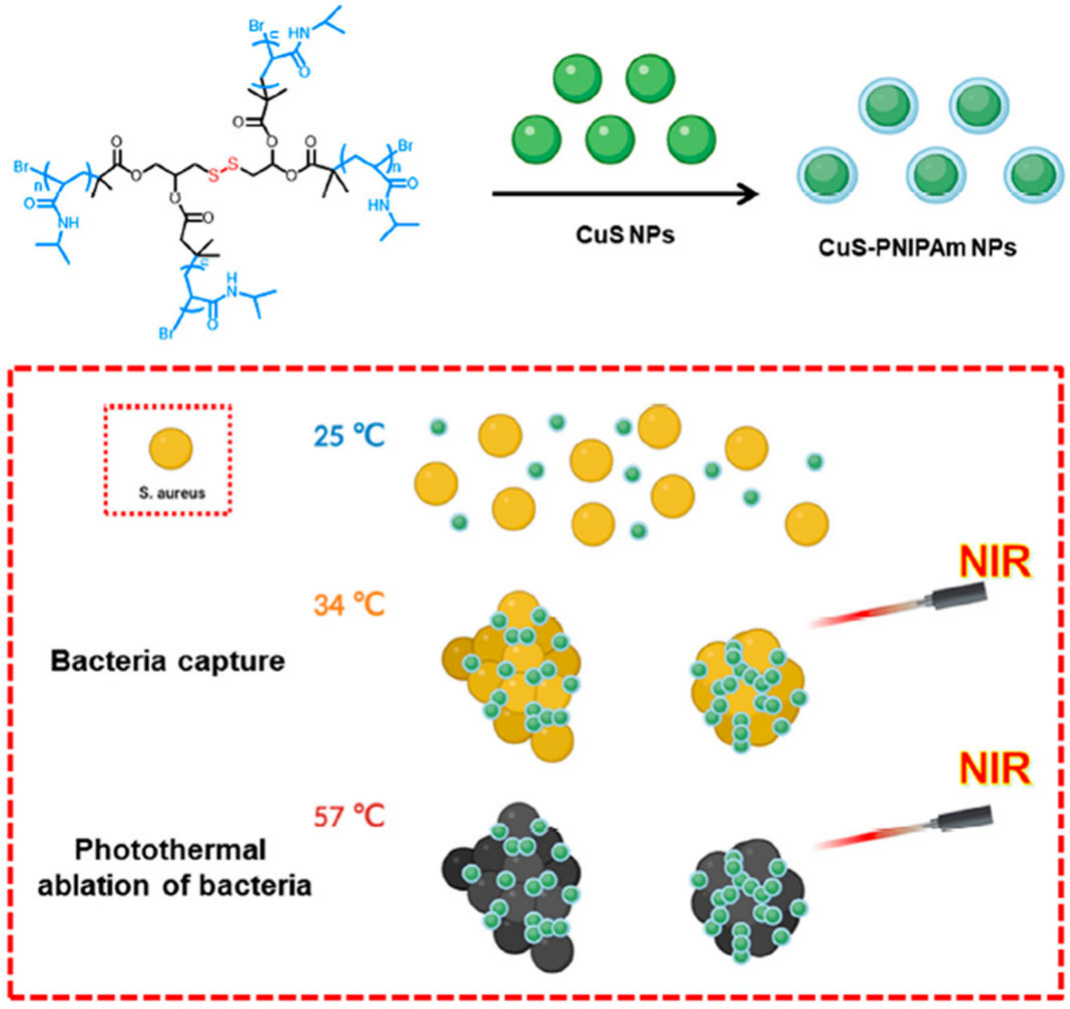
Schematic illustration of CuS-PNIPAm NPs preparation and capturing bacteria at above 34°C and effectively killing the bacteria under NIR irradiation.

In order to obtain CuS NPs with double effects of infected skin treatment and skin wounds regeneration acceleration, CuS NPs, shield by a four-arm temperature sensitive PNIPAm (4sPNIPAm), were fabricated to endow the PTT agent with the ability to initiatively capture/kill bacteria and with good compatibility, as illustrated in Fig. 1. Notably, this particular molecular structure is designed to improve the modification of the thermosensitive polymer on the surface of the NPs. 4sPNIPAm was firstly prepared by ATRP using initiator 4 s-Br containing disulfide bonds (Supplementary Fig. S1). The molecular weight of the synthesized 4sPNIPAm is about 9013.52 by NMR (Supplementary Fig. S2). The polymer is temperature-sensitive [31] and might coordinate with metal ions via the interaction between metal ions and disulfides [32].

**Figure 2.**
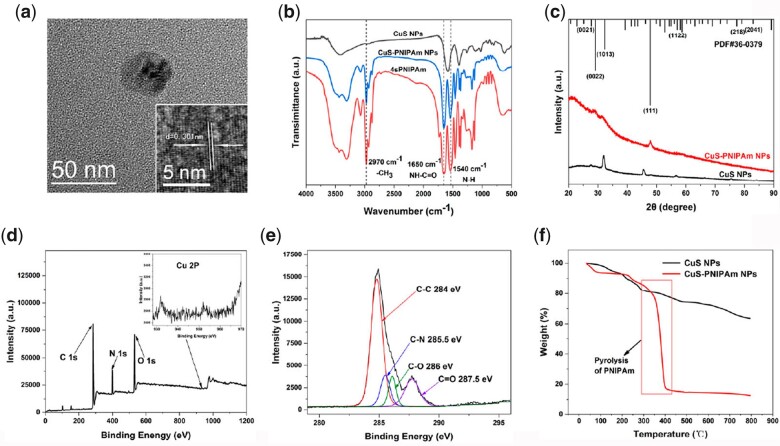
Characterization of CuS-PNIPAm NPs. (**a**) TEM image and high-resolution TEM image (inside). (**b**) FTIR spectra of CuS NPs, PNIPAm and CuS-PNIPAm NPs. (**c**) XRD spectra of CuS NPs and CuS-PNIPAm NPs. (**d**) XPS survey scan and (**e**) C 1 s spectrum of CuS-PNIPAm NPs. (**f**) TGA curves of CuS NPs and CuS-PNIPAm NPs.

4sPNIPAm was added into the newly prepared solution of Cit-CuS NPs (hereinafter Cit-CuS NPs was referred to as CuS NPs) and stirred for 12 h to obtain CuS-PNIPAm NPs. The diameter for CuS-PNIPAm NPs as shown in Fig. 2a is around 36 nm, coincident with the result of DLS (Supplementary Fig. S3). The average diameter is larger than that of CuS NPs (Supplementary Fig. S4), and increases approximately by 13 nm after 4sPNIPAm modification. The temperature-sensitive polymer can effectively modify the surface component of CuS NPs through the strong interaction between CuS NPs and 4sPNIPAm owing to not only the property of PNIPAm itself, but also the architecture of four arms and the composition of disulfide bond in the molecular structure. The DLS results of PNIPAm and CuS NPs mixed with PNIPAm proves the assumption (Supplementary Fig. S3). HRTEM image of CuS-PNIPAm NPs shows that the spacing between the lattice fringes of CuS-PNIPAm NPs on the interface is 0.301 nm.

The surface chemical composition of CuS-PNIPAm NPs was analyzed by Fourier transform infrared spectroscopy (FTIR) and X-ray photoelectron spectroscopy. Figure 2b shows that the peaks at 2970, 1650 and 1540 cm^−1^ appear, which are assigned to the characteristic peaks of PNIPAm, and correspond to -CH_3_, NH-C = O, N-H, respectively. In the X-ray diffraction spectrum, there are four main XRD peaks ranging from 20° to 90° for CuS NPs and CuS-PNIPAm NPs (Fig. 2c), which can match the standard diffraction pattern of Cu_9_S_8_ in the database of the international center for diffraction data.

The XPS survey scan for CuS-PNIPAm NPs is shown in Fig. 2d. The signal from 2P of copper is detectable, alike to CuS NPs (Supplementary Fig. S5), and the proportions of C, N and O are similar to those of PNIPAm. The high-resolution C 1 s spectrum of CuS-PNIPAm NPs can be fitted into the four characteristic signals of C-C, C-N, C = O and C-O (Fig. 2e). In comparison with the C 1 s spectra of CuS NPs and 4sPNIPAm (Supplementary Figs S5 and S6), there exist both PNIPAm and trisodium citrate on the surface of CuS-PNIPAm NPs. The TGA curves of CuS NPs and CuS-PNIPAm NPs indicate their pyrolysis from the mass loss between 300°C and 390°C, which can specific give the weight ratio of 4sPNIPAm (≈87.39 wt%) in CuS-PNIPAm NPs (Fig. 2f).

Among a variety of bio-safety and highly hydrophilic polymers used in the modification of NPs, PEG and its derivatives are the most common ones [33], since they have the lowest protein or cellular adsorption [34], and have been used in various clinical applications. As a contrast material, CuS NPs modified by HS-PEG (hereinafter referred to as CuS-PEG NPs) were prepared through the coordination of thiol and copper ion, and the main characteristics of CuS-PEG NPs are shown in Supplementary Figs S7–S9.

### Photothermal property of CuS-PNIPAm NPs

**Figure 3.**
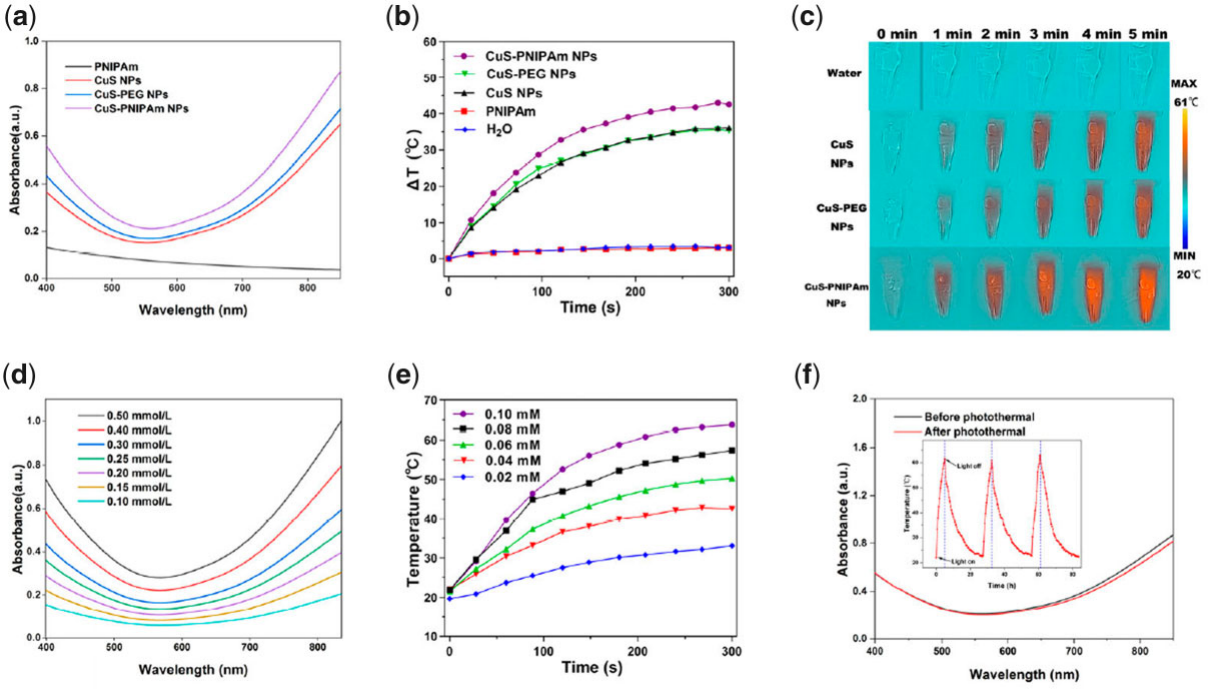
UV-Vis absorbance and photothermal property of CuS-PNIPAm NPs. **(a**) UV-vis absorbance spectra of 4sPNIPAm, CuS NPs, CuS-PEG NPs and CuS-PNIPAm NPs (0.5 mM). (**b**) Temperature evolution profiles and (**c**) photothermal images of CuS NPs, CuS-PEG NPs, CuS-PNIPAm NPs, 4sPNIPAm and H_2_O upon NIR laser irradiation. (**d**) UV-vis absorbance spectra and (**e**) temperature changes of CuS-PNIPAm NPs with different concentrations. (**f**) UV-vis absorbance spectra of CuS-PNIPAm NPs before and after undergoing NIR irradiation. The inset diagram of (f) is the temperature profile of CuS-PNIPAm NPs undergoing three repetitive irradiation for 5 min with 808 nm, 2 W/cm^2^.

Photothermal NPs should effectively respond to NIR light at 808 nm [35, 36]. The photothermal effect of CuS-NPs is dominantly attributed to localized surface plasmon resonance absorption caused by free holes originating from cation vacancies [37]. The UV-vis spectra of CuS-NPs, CuS-PEG NPs and CuS-PNIPAm NPs between 400 and 900 nm were measured (Fig. 3a). All of the NPs show obvious NIR absorbance (700–900 nm). Interestingly, although PNIPAm has almost no absorption in entire UV-vis interval, the absorbance of CuS-PNIPAm NPs is significantly higher than that of the other two kinds of NPs in the NIR region.

**Figure:**
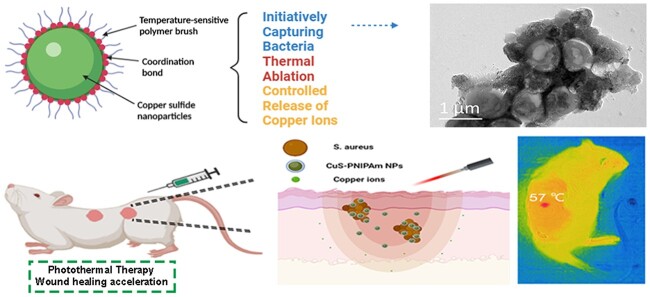


Temperature enhancement of CuS-PNIPAm NPs is the biggest under NIR irradiation (808 nm, 2 W cm^−2^, 300 s), up to 43°C for 0.1 mM solution (200 μl), meanwhile only 36°C and 34°C for CuS NPs and CuS-PEG NPs in the same condition, respectively (Fig. 3b). The thermal images (Fig. 3c) are consistent with the above phenomena. The superior photothermal property of CuS-PNIPAm NPs may be caused by PNIPAm via the enhanced refraction times of light on the surface of copper sulfide NPs, leading to the enhancement of absorption, according to the reference [38]. In Supplementary Fig. S10, UV-vis absorbance of the mixture of CuS NPs and 4sPNIPAm at 808 nm displays imperceptible variation with CuS NPs, meantime, is markedly lower than that of CuS-PNIPAm NPs, revealing that the successful package of 4sPNIPAm on CuS NPs indeed boosts photothermal effect.

The absorption intensity of CuS-PNIPAm NPs in the NIR region increases with the increase of copper concentration in NPs (Fig. 3d). Similarly, the temperature increase under 808 nm irradiation displays a dependency on NPs concentration. For example, the temperature ascends by 37.4°C and 30.3°C for 0.08 mM and 0.06 of CuS-PNIPAm NPs under NIR irradiation for 5 min. The photothermal behavior of CuS-PNIPAm NPs is not only prominent, but also sustainable. The PTT effect of CuS-PNIPAm NPs does not show any decrease within three cycles (the inset of Fig. 3f). The sustainable photothermal property of CuS-PNIPAm NPs is rooted from the almost unchanged structure of the NPs, as the ultraviolet absorption spectrum shows that there is only a slight attenuation of CuS-PNIPAm NPs (Fig. 3f). In a word, all the data suggest the excellent photothermal transformation of CuS-PNIPAm NPs.

### Stability improvement of NPs and the controlled release of copper ions

**Figure 4.**
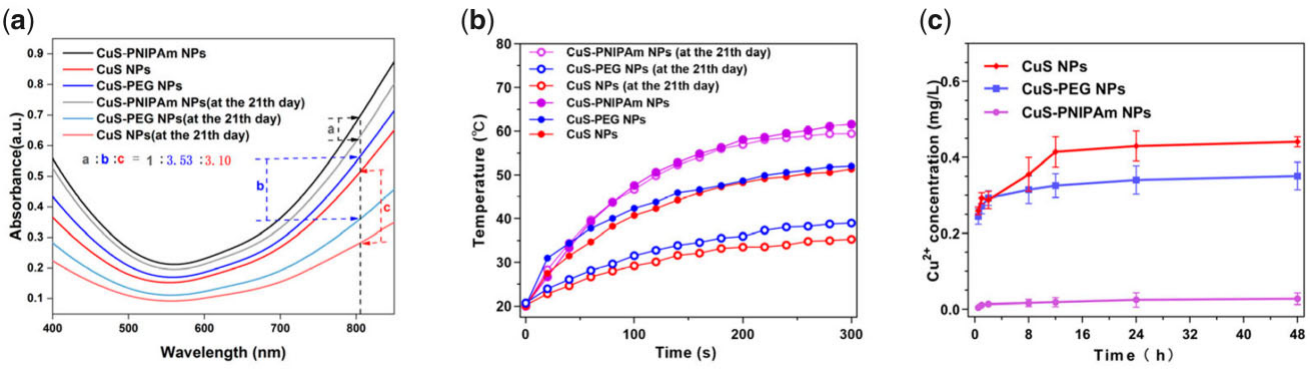
Stability of NPs and the controlled release of copper ions. (**a**) UV-vis absorbance spectra and (**b**) temperature evolution profiles under NIR laser irradiation of fresh and stored CuS NPs, CuS-PEG NPs and CuS-PNIPAm NPs (0.5 mM) for 21 days. (**c**) Cu^2+^ cumulative release curves in PBS of CuS NPs, CuS-PEG NPs and CuS-PNIPAm NPs at 37°C in 48 h (*n* = 3).

As shown in Fig. 4a, the UV-vis spectra of the fresh and stored NPs indicate that the absorption of the three NPs decreases after storage. The decreasing amplitude of the absorption at 808 nm is the largest for CuS-PEG NPs, CuS NPs followed, and the lowest for CuS-PNIPAm NPs. The ratios of the decreased amplitude of 21 days among the three NPs are 3.53:3.10:1. In other words, CuS-PNIPAm NPs present the best storage stability. The stability improvement of CuS NPs by 4sPNIPAm is also reflected in photothermal test (Fig. 4b). At the 21st day after 300 s laser irradiation, the photothermal effect of CuS-PNIPAm NPs remains almost the same as in the beginning while the maximum temperature for CuS-NPs and CuS-PEG NPs decreases by 21°C and 17°C, respectively. The difference in the photothermal effect can be explained by the release of copper ions of the NPs, as measured by ICP-AES. As shown in Fig. 4c, the concentration of the leaching copper ions in PBS for CuS-PNIPAm NPs in 48 h remains to be at quite low level. In the meantime, the concentration of the leaching copper ions is up to 0.25–0.27 and 0.22–0.26 mM for CuS NPs and CuS-PEG NPs even in 0.5 h, respectively. Therefore, the modification on CuS NPs surface by 4sPNIPAm significantly enhances the stability of storage and photocorrosion.

The super photothermal effect and stability of CuS-PNIPAm NPs compared with CuS NPs and CuS-PEG NPs are reasonably speculated as follows. 4sPNIPAm encapsulates CuS NPs without affecting the dispersion of the NPs in water alike the surfactant with similar structure of hydrophobic alkyl and hydrophilic short-chain poly (ethylene glycol) [1]. More importantly, the amide group is a ligand for metal ions, donating from the oxygen atom, whose electron density is augmented by N lone pair resonance [39]. Consequently, some of the copper ions released from the NPs under NIR irradiation are then chelated by amide groups of PNIPAm on the surface of the NPs, and are kept in the NPs. That leads to the limited rise of copper ions in the solution under NIR irradiation or during storage. The advantages of the controlled release behavior are obvious. On one hand, it slows down the deconstruction of the NPs, leading to the improved stability of NPs. On the other hand, it keeps the concentration of copper ions at a relatively low level, 0.07–0.09 mM in 0.5 h under NIR irradiation for 5 min (Supplementary Fig. S11), which is beneficial for angiogenesis during wound healing [15].

### Heat- and photothermal-induced aggregations of NPs-NPs and NPs–bacteria

**Figure 5.**
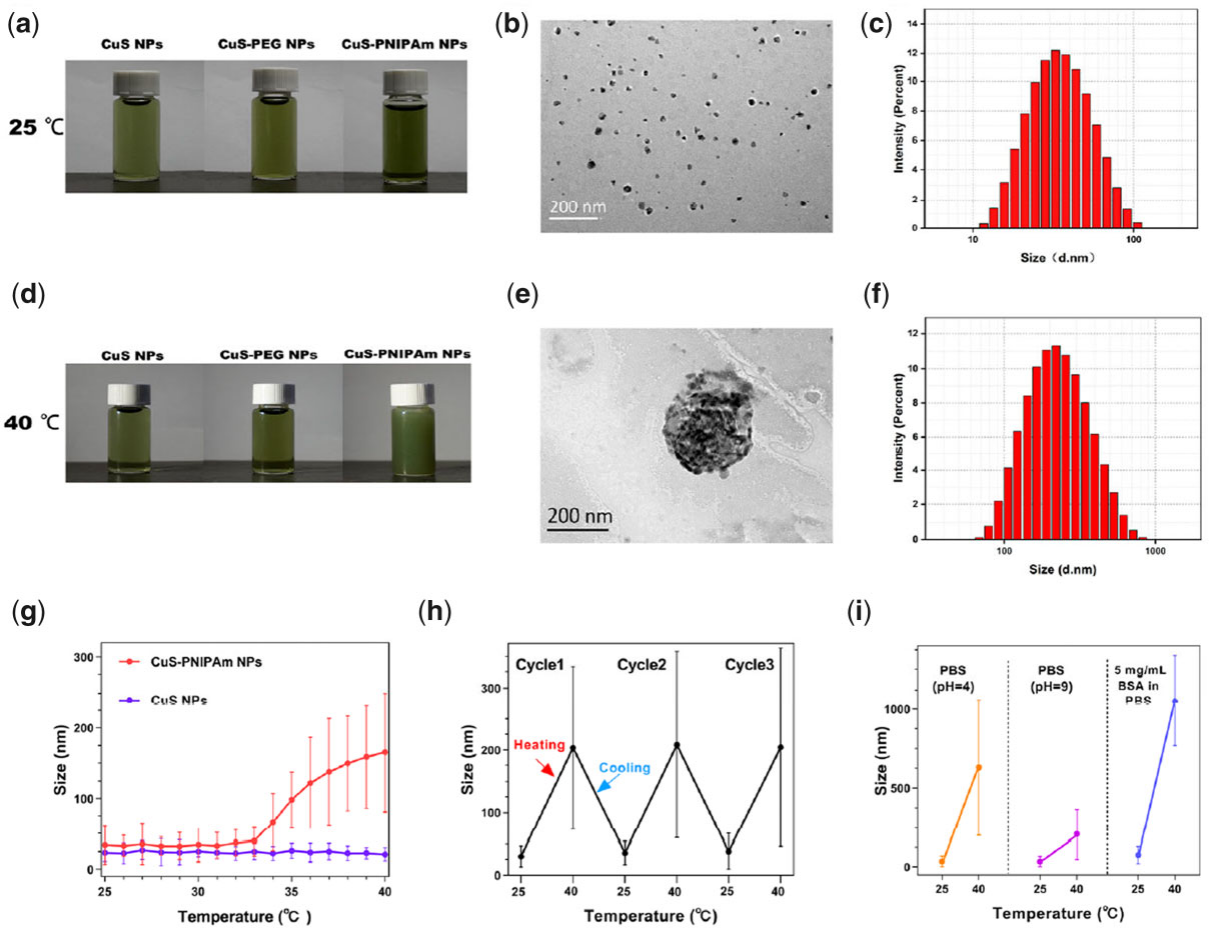
Heat- and photothermal-induced aggregations of CuS-PNIPAm NPs. The photos of CuS NPs, CuS-PEG NPs and CuS-PNIPAm NPs solution at 25°C (**a**) and at 40°C (**d**). TEM images and particle size distribution of CuS-PNIPAm NPs at 25°C (**b, c**) and at 40°C (**e, f**). The sizes of CuS NPs and CuS-PNIPAm NPs at 25°C–40°C (**g**). the size changes of CuS-PNIPAm NPs at 25–40°C for three cycles (**h**) and under different media (**i**).

As shown in Fig. 5a–c, the macroscopic appearance and the results of transmission electron microscope (TEM) and DLS for CuS-PNIPAm NPs show good dispersibility in water at low temperature. Meanwhile, the aggregates beyond 100 nm occur at 40°C when temperature exceeds the LCST of PNIPAm as shown in Fig. 5d–f, implying transformation of PNIPAm on the surface of the NPs from hydrophilic to hydrophobic status. However, CuS NPs and CuS-PEG NPs do not exhibit such thermal response behavior. As revealed in Fig. 5g and Supplementary Fig. S12, the particle sizes of CuS NPs and CuS-PEG NPs measured by DLS are quite constant in the range from 25 to 40°C, which is quite different from that of CuS-PNIPAm NPs. The particle sizes of CuS-PNIPAm NPs continually increase when the temperature is above 34°C. The stable thermal sensitivity of CuS-PNIPAm NPs between 25°C and 40°C is also proved, as shown in Fig. 5h. Furthermore, the thermo-induced aggregations of CuS-PNIPAm NPs also occur in different media including acidic (pH = 4), basic (pH = 9) solution and 5 mg ml^−1^ BSA in phosphate buffered saline (PBS), as shown in the Fig. 5i. It is found that CuS-PNIPAm NPs can perform the best thermal sensitivity under PBS with BSA. That means there still exists thermo-induced status transformation of CuS-PNIPAm NPs in different medium conditions.

**Figure 6.**
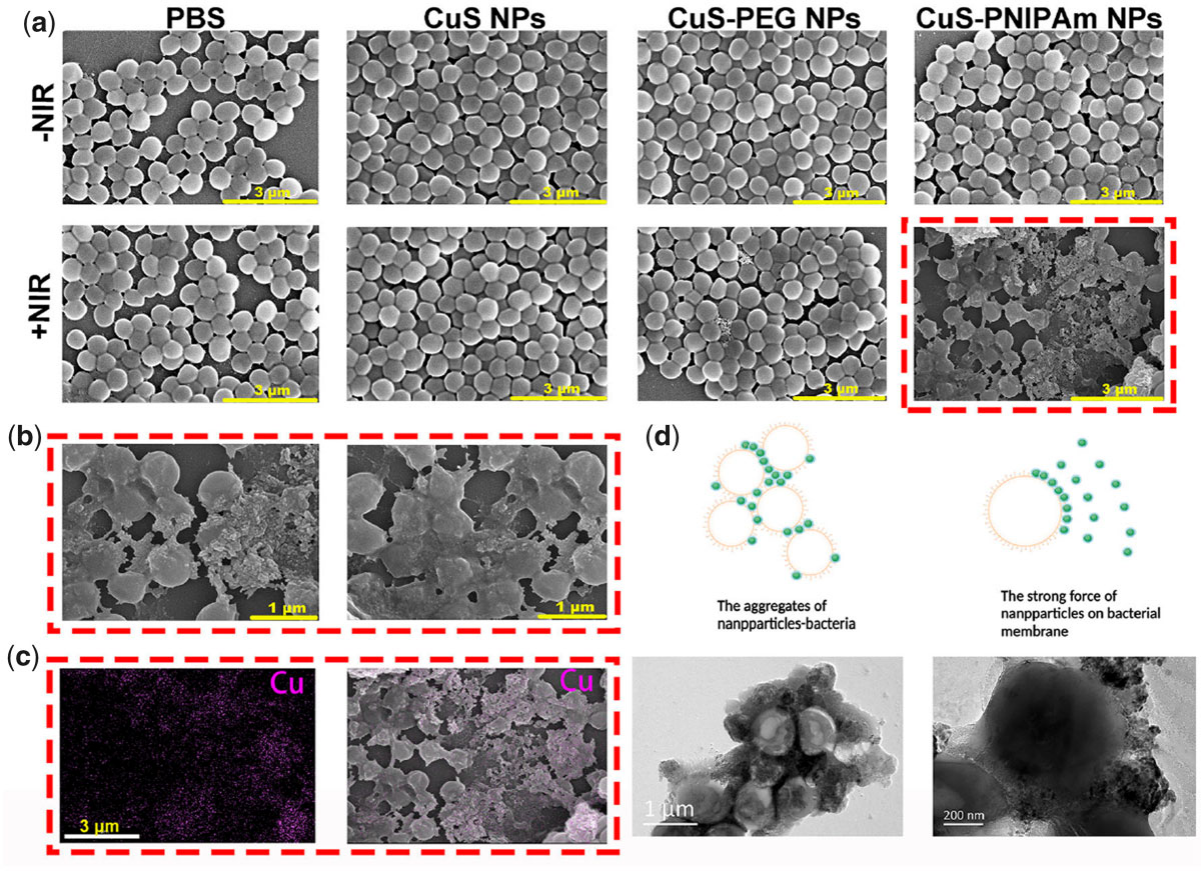
Effect of NIR irradiation on bacteria in NPs solutions. (**a**) SEM images of *S.aureus* without or with NIR irradiation in different solutions. (**b**) Local amplification and (**c**) EDAX mapping images of copper ions for SEM image of *S.aureus* in CuS-PNIPAm NPs with NIR irradiation. (**d**) TEM images of the aggregates of NPs–NPs and NPs–bacteria induced by NIR irradiation. NIR irradiation proceeded for 5 min by a laser with 808 nm, 2 W/cm^2^.

In order to examine the feasibility of CuS-PNIPAm NPs’s under NIR irradiation through photothermal transformation to capture and kill bacteria, photothermal-induced aggregations and bacteria killing were studied. As shown in Fig. 6a, *S.aureus* added into different solutions without irradiation show normal spherical morphology, and the NPs disperse well due to their hydrophilic property. Once upon NIR irradiation, *S.aureus* in the NP solutions shrink and the morphology collapse appear for some *S.aureus* owing to photothermal effect, different from PBS group. It should be noted that *S.aureus* of CuS-PNIPAm NPs group are totally different from those in other groups, i.e. almost all of the *S.aureu*s display obviously the aggregates with collapsed morphologies seemingly through some stuff. The wizened and deformed *S.aureus* are clearly verified by SEM images (Fig. 6b), implying that the *S.aureus* are dead. The *E.coli* added into different solutions with and without irradiation demonstrate much the same (Supplementary Fig. S13).

In order to find the reason of the more excellent photothermal effect for CuS-PNIPAm NPs than CuS NPs and CuS-PEG NPs against bacteria, EDAX mapping of copper ions for *S.aureus* added into CuS-PNIPAm NPs with NIR irradiation were imaged (Fig. 6c). From the overlay images, we can see that the distribution of copper ions is not homogeneous, unlike in CuS NPs and CuS-PEG group (Supplementary Fig. S14) and is on the surfaces of bacteria and concentrates on the intermediate regions of the conjoint bacteria. The heterogeneous distribution of copper ions is rooted from the aggregation of NPs or NPs –bacteria. The local images of TEM shown in Fig. 6d and Supplementary Fig. S13c provide confirmative evidence that the strong interaction between CuS-PNIPAm NPs and bacteria arise upon NIR irradiation.

Since the zeta potentials of the NPs are negative (Supplementary Fig. S9), the interaction between NPs and bacteria does not stem from electrostatic effect. Under NIR irradiation, all of the NPs can generate heat owing to photothermal effect. The heat leads to the increasement of temperature to above the LCST of PNIPAm. At this moment, PNIPAm chains on the surface of the NPs convert to hydrophobicity, which results in the NPs adhering each other or to the surface of bacteria. Therefore, it is supposed that the hydrophobic interaction plays an important role between CuS-PNIPAm NPs and bacterial membranes. The adhered CuS-PNIPAm NPs on bacteria further produce heat under NIR to efficiently destroy the cell membrane of bacteria. Taken together, whether it’s *S.aureus* (Gram-positive) or *E.coli* (Gram-negative), CuS-PNIPAm NPs can effectively aggregate and produce heat on bacterial surfaces, leading to the damage and atrophy of bacterial membranes. All of the data confirm the thermo-sensitive trapping behavior and the broad-spectrum antibacterial action of CuS-PNIPAm NPs.

### Antibacterial performances

As studied above, CuS-PNIPAm NPs exhibit the ability to initiatively capture bacteria under NIR irradiation. Thus, we may speculate that CuS-PNIPAm NPs will present an efficient photothermal killing bacteria action.

**Figure 7.**
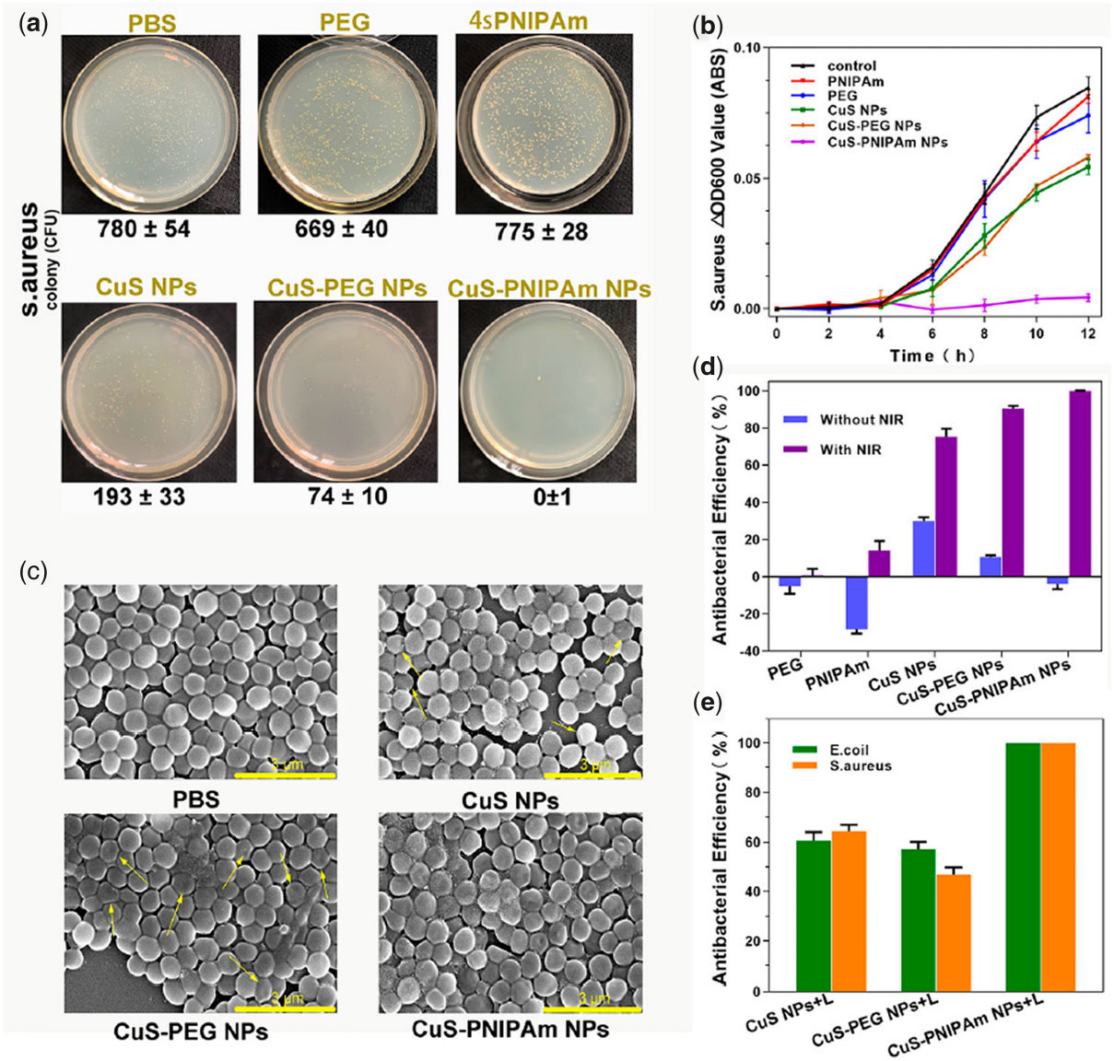
Antibacterial effect of PTT *in vitro*. (**a**) Photographs on the LB ager plates and (**b**) OD 600 values and (**c**) SEM images for *S.aureus* in different solutions with NIR irradiation. Antibacterial efficiencies against *S.aureus* with and without NIR irradiation (**d**) and against *S.aureus* and *E.coli* (**e**) under NIR irradiation. The concentration for different solutions is 0.08 mM. NIR irradiation proceeded for 5 min by a laser with 808 nm, 2 W/cm^2^.

Figure 7a shows the results of 24 h colony cultivation in agar plates for *S.aureus* after irradiation. The killing efficiency of CuS-PNIPAm NPs against *S.aureus* is nearly 100% and superior to all the control groups, which is similar to *E.coli* (Supplementary Fig. S15a). The OD_600_ values of the irradiated bacteria at 12 h cultivation display the same tendency as the results of agar plate counting (Fig. 7b and Supplementary Fig. S15c). The images of the bacteria obtained by SEM images are shown in Fig. 7c and Supplementary Fig. S15b. With NIR irradiation, *S.aureus* and *E.coli* mixed with NPs appear with comprehensive merged and collapsed membranes, which are different from the bacteria with smooth clear edges in PBS group. Especially, the deformed bacteria in CuS-PNIPAm NPs group are more than in CuS NPs and CuS-PEG NPs, which is rooted from superior photothermal effect of CuS-PNIPAm NPs directly applying to the surface of the cell membrane of the bacteria.

Comparing the antibacterial ratios against *S.aureus* (Fig. 7d) and *E.coli* (Supplementary Fig. S15d) with and without NIR irradiation, the photothermal antibacterial performance of CuS-PNIPAm NPs under irradiation is perfect, i.e. the killing efficiency to either *S.aureus* or *E.coli* is up to 100%, while for CuS NPs or CuS-PEG NPs are <60%, as shown in Fig. 7e. On the whole, CuS-PNIPAm NPs possess superior antibacterial activity over the other two NPs, which is attributed to the role of 4sPNIPAm in improving antimicrobial efficiency because of the aggregate formation of NPs–NPs and NPs–bacteria under the photothermal effect.

Furthermore, the influence of sample concentration and irradiation time on bacterial killing efficiency against *S.aureus* and *E.coli* was also detected (Supplementary Figs S16 and S17). The bacterial killing efficiency enhances with the increase of irradiation time and sample concentration due to the increase of generation heat. Therefore, it can be concluded that the antibacterial activity of CuS-PNIPAm NPs can be facilely controlled and manipulated as the application environment changes.

### Toxic effect

**Figure 8.**
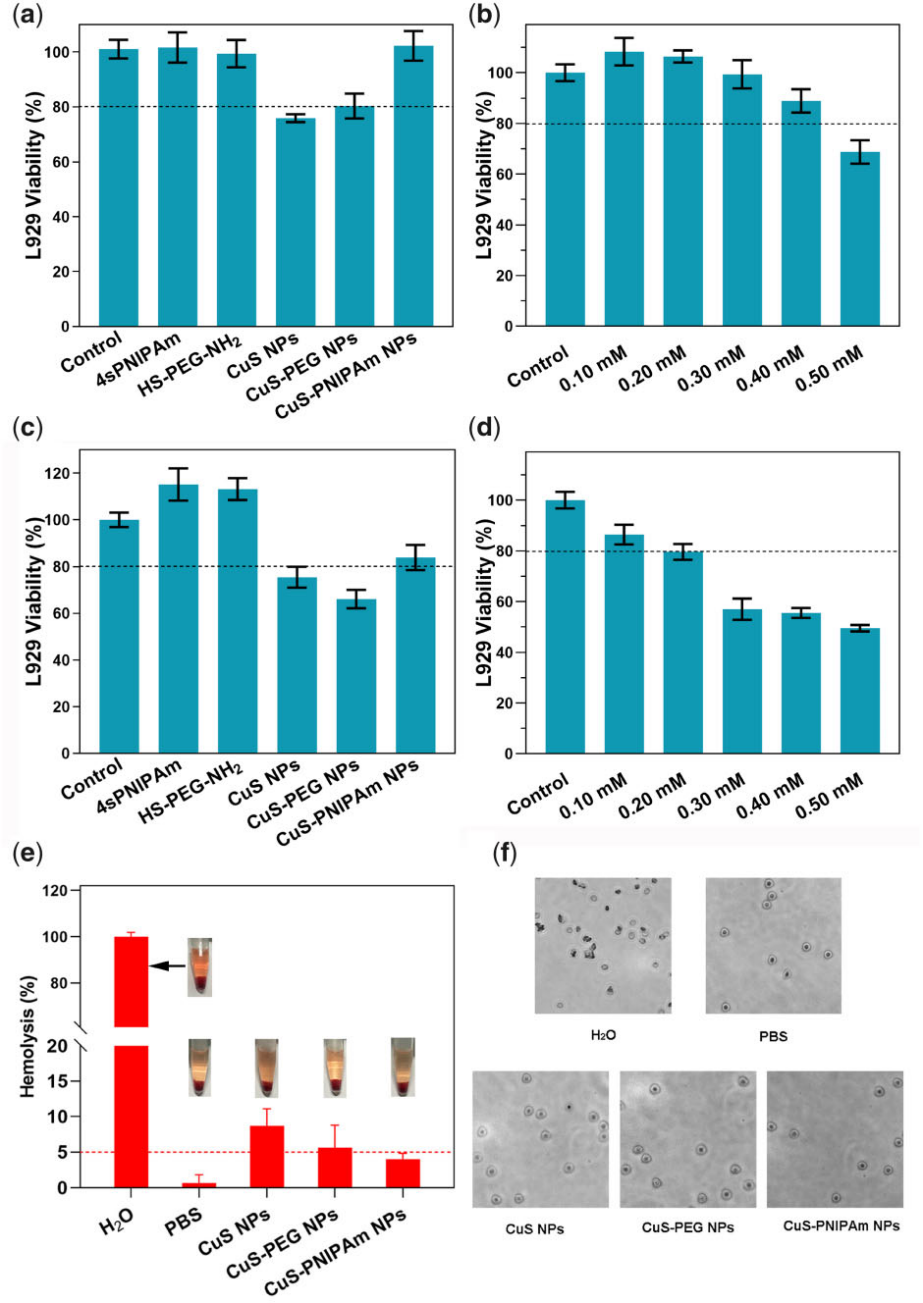
Cytotoxicity *in vitro*. Viability of L929 cells co-cultured with different solutions (0.2 mM) (**a, c**) and at different concentrations of CuS-PNIPAm NPs (0.1–0.5 mM) (**b, d**) without (**a, b**) and with NIR irradiation (**c, d**). NIR irradiation proceeded for 1 min by a laser with 808 nm, 2 W/cm^2^. (**e**) Photographs and hemolysis ratio of RBCs for H_2_O (positive control), PBS (negative control), CuS NPs, CuS-PEG NPs and CuS-PNIPAm NPs (*n* = 3). (**f**) Morphology of RBCs after hemolysis assay.

The NPs of copper sulfides may produce dual toxic effect of NPs and copper ions. The viability of L929 cells co-cultured with the samples is shown in Fig. 8a. There is no significant difference in the cell viability between CuS-PNIPAm NPs and control groups (blank, 4sPNIPAm and HS-PEG-NH_2_ solution). The survival rate in CuS-PNIPAm NPs is high, indicating that CuS-PNIPAm NPs have good biocompatibility without NIR irradiation.

To evaluate the potential toxicity of CuS-PNIPAm NPs to cells under laser irradiation, the viability of L929 cells, hemolysis ratio and morphology of RBCs co-cultured with the samples under laser irradiation also were tested (Fig. 8c–f). When the NPs concentration is at 0.2 mM, the cell survival rates for all samples are over 75% (Fig. 8c). This indicates that although the temperature produced by the NPs under irradiation is up to 57°C ultimately, the viability of normal cells does not sharply decrease, since that hyperpyrexia can only last for a short time (<150 s), which is not enough to cause damage to normal cells. The survival rate change in CuS-PNIPAm NPs with concentration also proves that CuS-PNIPAm NPs with concentration <0.2 mM under 5 min NIR irradiation have no cytotoxicity (Fig. 8d). The different effect of CuS-PNIPAm NPs under NIR irradiation to different cells may be rooted from the difference of cell surface structures and properties between healthy cells and bacteria.

The hemolysis ratio of CuS-PNIPAm NPs is <5% (Fig. 8e). The morphology of RBCs in CuS-PNIPAm groups is unchanged after the experiment (Fig. 8f). On the contrary, CuS NPs and CuS-PEG NPs show hemolysis at 0.2 mM concentration, with hemolysis rates of 8.71% and 5.62%, respectively. Thus, CuS-PNIPAm NPs possess blood compatibility. This may be due to the low release of copper ions under the same concentration of NPs. In summary, CuS-PNIPAm NPs can act safely as a photothermal agent without worrying about toxic effect.

### *In vivo* wound healing performance

**Figure 9.**
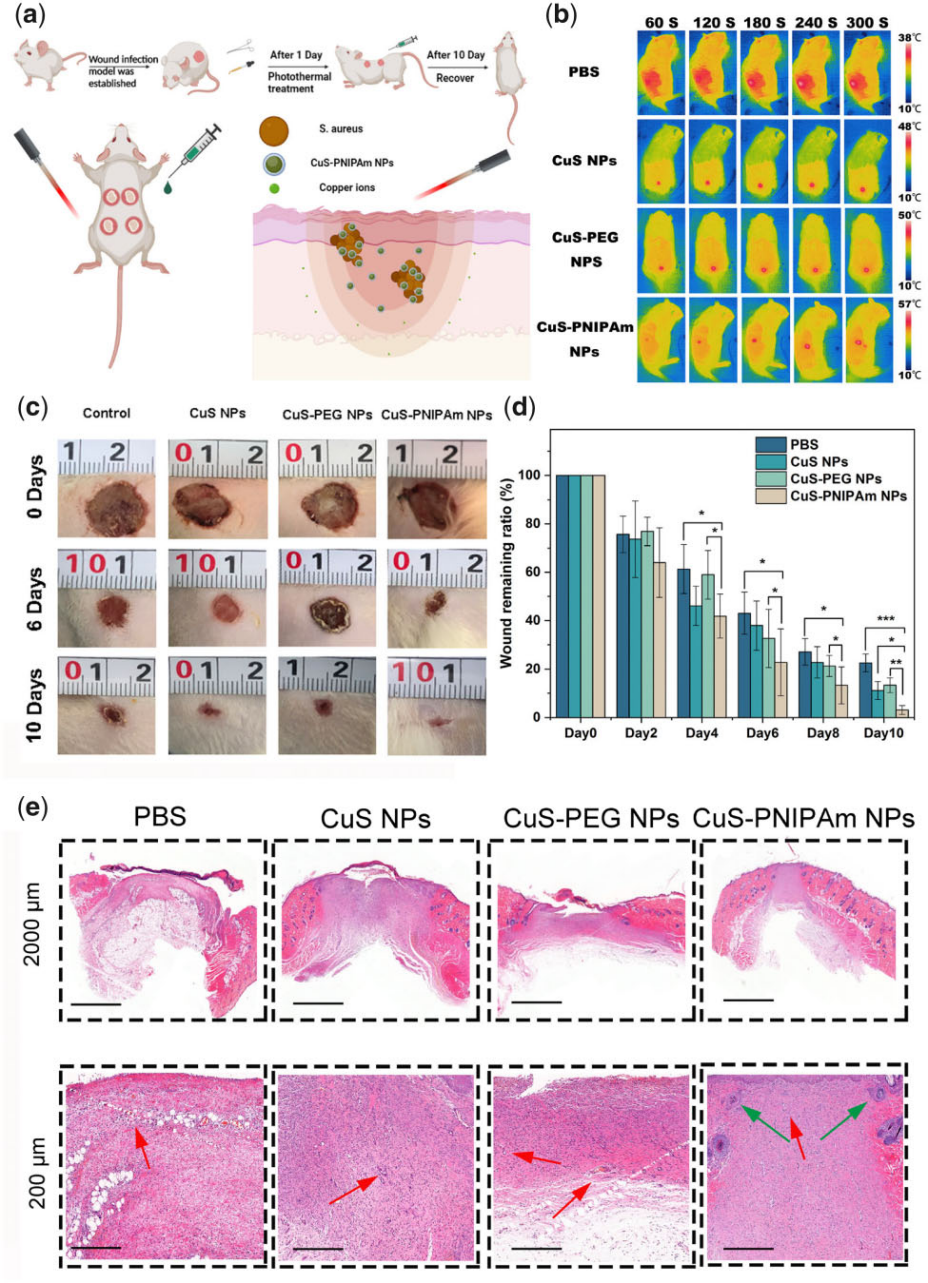
Antibacterial effect of CuS NPs PTT *in vivo*. (**a**) Schematic illustration of establishment and treatment of full-skin infected wounds. (**b**) Photothermal images for the mouse skin under NIR irradiation. (**c**) Wound photographs taken on the 0th, 6th and 10th day and (**d**) wound remaining ratios of the full-skin infected rats (*n* = 5). (**e**) H&E staining images of the rat dermal wounds on the 10th day of treatment.

A full-skin wound infection model on SD rats was established to evaluate the wound healing efficacy of CuS-PNIPAm NPs *in vivo*, flow diagram and PTT imaging is shown in Fig. 9a and b. According to the experimental results, the PTT temperature in the CuS-PNIPAm NPs group increases to 57°C in the end of NIR irradiation for 300 s (Fig. 9b). However, the temperature increase displays a dependency with irradiation time (Supplementary Fig. S18). That is, the temperature at the treatment site may lower than 45°C for CuS-PNIPAm NPs at 0.2 mM in almost half of the time (144 s), which does cause obvious damage to normal tissues. The change images of the wounds in different days are shown in Fig. 9c. The ratio of wound remaining areas in appearance of CuS-PNIPAm NPs group is smaller than those of the other groups during the whole treatment (Fig. 9d). On the sixth day, the remaining ratio of wound area of PBS, CuS NPs, CuS-PEG NPs, and CuS-PNIPAm NPs groups is 43.03%, 37.98%, 32.65% and 22.75%, respectively, starting to show significant smaller for CuS-PNIPAm NPs group than that of the others (Fig. 9d). In addition, the closure wound is observed for CuS-PNIPAm group on the 10th day, whereas the remaining ratios of wound areas for PBS, CuS NPs and CuS-PEG NPs groups are still ∼22.56%, 11.06% and 13.28%, respectively. Thus, the ability of CuS-PNIPAm NPs to promote the healing of infected wounds is significantly higher than that of other groups.

In H&E staining images, the new epidermis formed in the wounds show different characteristics, and the regenerated hair follicles (green arrows) can be distinct observed only in the CuS-PNIPAm NPs groups (Fig. 9e). The others remain at the stage of capillaries (red arrows).

Conclusively, in comparison with natural recovery, the bacteria in infected wounds can be efficiently killed, and the wound healing obviously accelerates for the groups treated by NPs upon NIR. The better effect of CuS-PNIPAm NPs than the other two NPs groups may be attributed to a combination of more thorough bacterial removal and safer copper ion concentrations. Therefore, CuS-PNIPAm NPs can achieve more efficient regeneration of infected wounds.

## Conclusions

In summary, we designed a 4sPNIPAm, and then used it to modify CuS NPs in order to obtain a biocompatible PTT agent with the ability to initiatively capture/kill bacteria. The prepared CuS-PNIPAm NPs are with heat and near-infrared light response. CuS-PNIPAm NPs present higher photothermal conversion ability and controlled release of copper ions owing to the interaction between 4sPNIPAm and copper ions in comparison with contrastive CuS-PEG NPs and CuS-NPs. In addition, CuS-PNIPAm NPs can aggregate at above 34°C and display stable thermal sensitivity between 25°C and 40°C due to the transformation from hydrophilic state to hydrophobic aggregation state of PNIPAm. The behavior endows CuS-PNIPAm NPs with the ability to capture bacteria by forming aggregates of NPs –bacteria. Furthermore, both *S.aureus* and *E.coli* can be completely killed upon NIR irradiation in minutes, validating the excellent activity of CuS-PNIPAm NPs against Gram-negative and Gram-positive bacteria. The release of copper ions from CuS-PNIPAm NPs is low, leading to their nontoxic effect. *In vivo*, the NPs can effectively kill the bacteria in the wounds and accelerate the process of wound repair. Overall, biocompatible CuS-PNIPAm NPs exhibit potential applications in antibacterial fields and regeneration of skin tissues.

## Supplementary Material

rbac026_Supplementary_Data
