# Supplementary material for: CuS-PNIPAm nanoparticles with the ability to initiatively capture bacteria for photothermal treatment of infected skin
Source: Regen Biomater. 2022 Apr 29;9:rbac026. doi: 10.1093/rb/rbac026 (PMC9128540; doi:10.1093/rb/rbac026)
Supplement: rbac026_Supplementary_Data [file rbac026_supplementary_data.docx]

**CuS-PNIPAm NPs with the Ability to Initiatively Capture Bacteria for Photothermal Treatment of Infected Skin**

Zizhen Wang^a^, Zishuo Hou^a^, Peiwen Wang^a^, Fan Chen*^a^ and Xianglin Luo *^ab^

*^a^* College of Polymer Science and Engineering, Sichuan University, Chengdu, People’s Republic of China.

*^b^* State Key Laboratory of Polymer Materials Engineering, Sichuan University, Chengdu, People’s Republic of China

* Corresponding authors.

E-mail Addresses： Luoxl@scu.edu.cn ( Xl. Luo)

**Support Information**





**Figure. S1.** The synthesis illustration of 4s-Br initiator and its ^1^H-NMR spectrum





**Figure. S2.** ^1^H-NMR spectrum of 4sPNIPAm


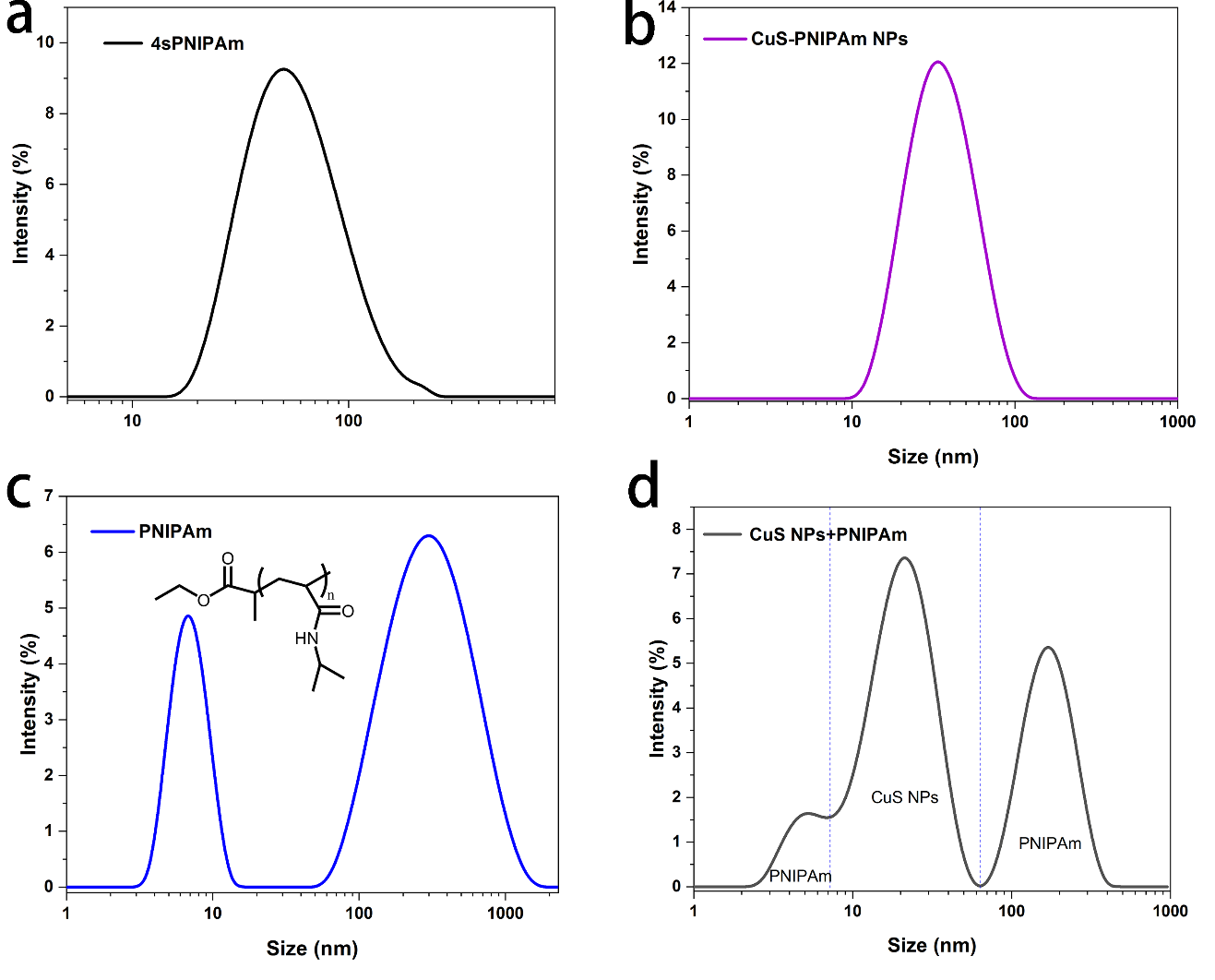


**Figure. S3.** Dynamic hydration diameter measured by DLS. (a) 4sPNIPAm NPs and (b) CuS NPs mixed 4sPNIPAm for 24 h stirring, (c) PNIPAm terminated in ethyl propionate and (d) CuS NPs mixed PNIPAm terminated in ethyl propionate for 24 h stirring.





**Figure. S4.** Dynamic hydration diameter of CuS NPs under 25 °C and 40 °C measured by DLS.


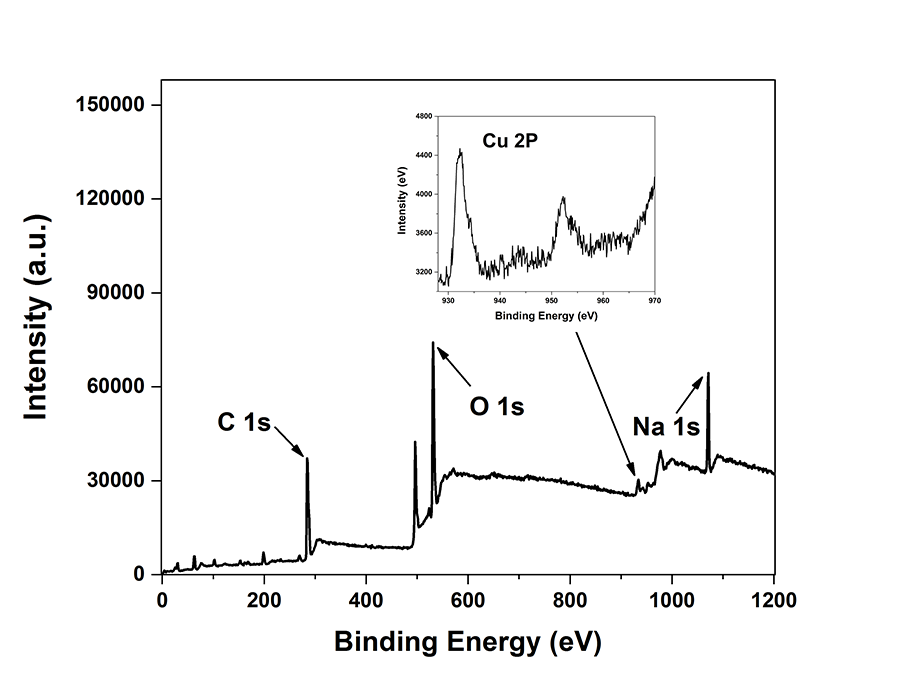





**Figure S5.** XPS survey scan (A) and C 1s spectrum (B) of CuS NPs








**Figure. S6.** XPS survey scan (A) and C 1s spectrum (B) of 4sPNIPAm





**Figure. S7.** DLS of CuS-PEG NPs under 25 °C and 40 °C


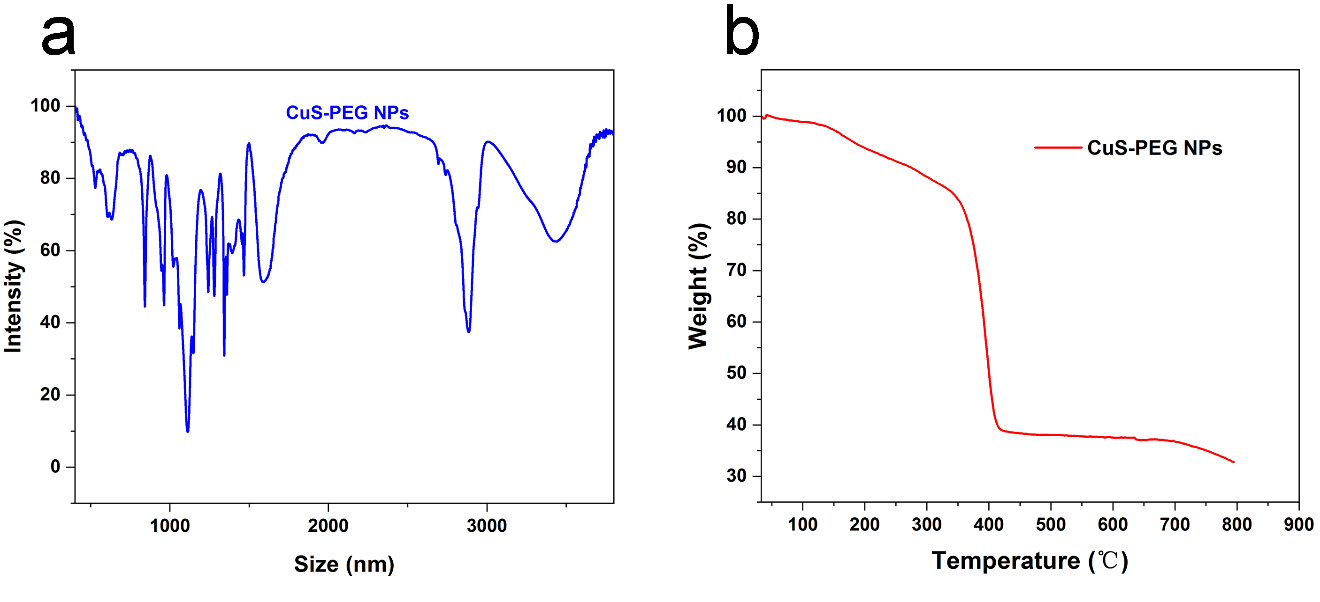


**Figure. S8.** (a) FTIR spectra of CuS-PEG NPs. (b) TGA curves of CuS-PEG NPs


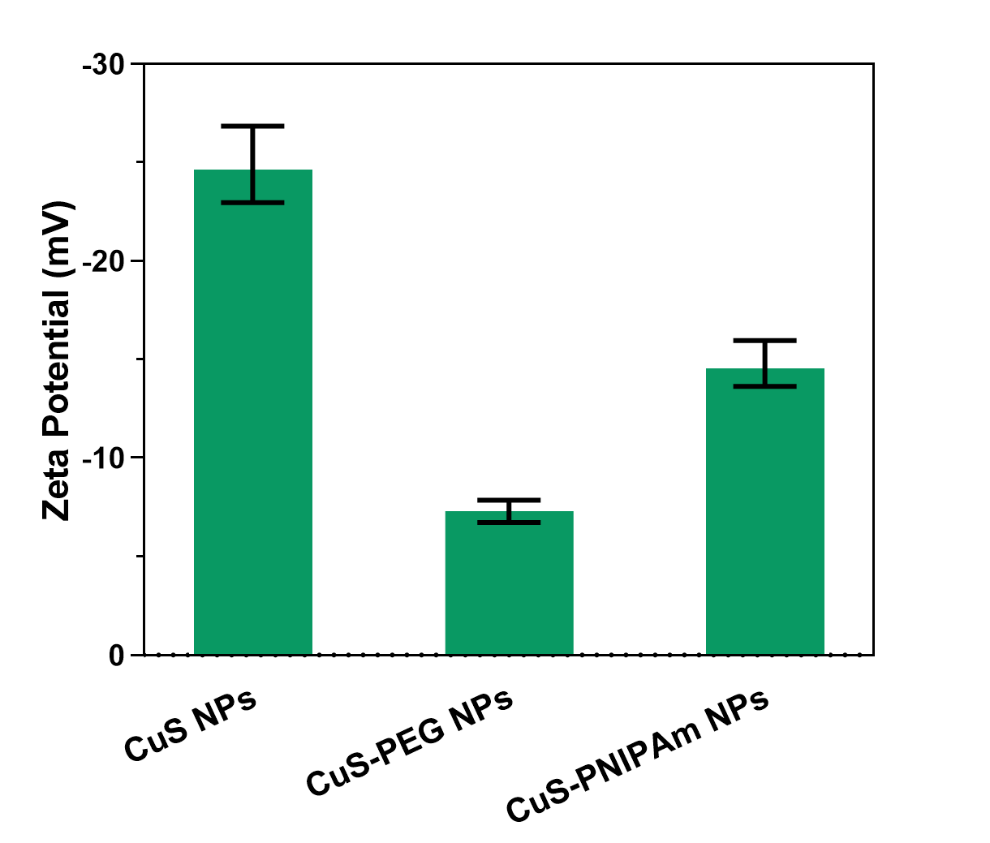


**Figure. S9.** The zeta potential of CuS NPs, CuS-PEG NPs and CuS-PNIPAm NPs.





**Fig. S10.** UV-vis absorbance spectra of CuS-PNIPAm NPs, CuS NPs, PNIPAm, CuS NPs+PNIPAm (0.5 mM). CuS NPs+PNIPAm means the mixture of CuS NPs and 4sPNIPAm for instantaneous measurement after being mixed.


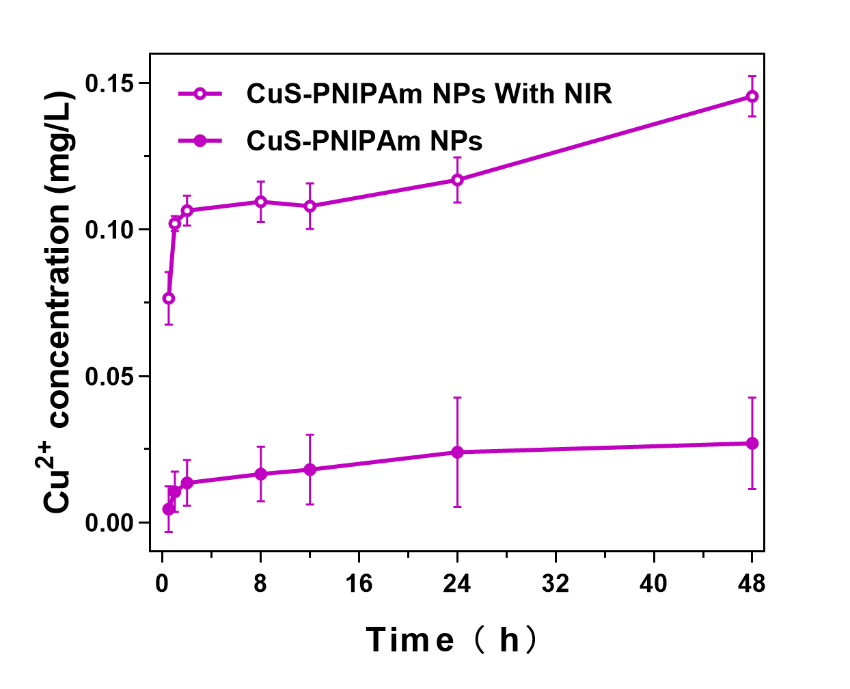


**Figure. S11.** Cu^2+^cumulative release curves of CuS-PNIPAm NPs (with and without NIR) in PBS at 37℃ for 48 h (n = 3). NIR irradiation conducts with 808 nm, 2 W /cm^2^ for 5 min.


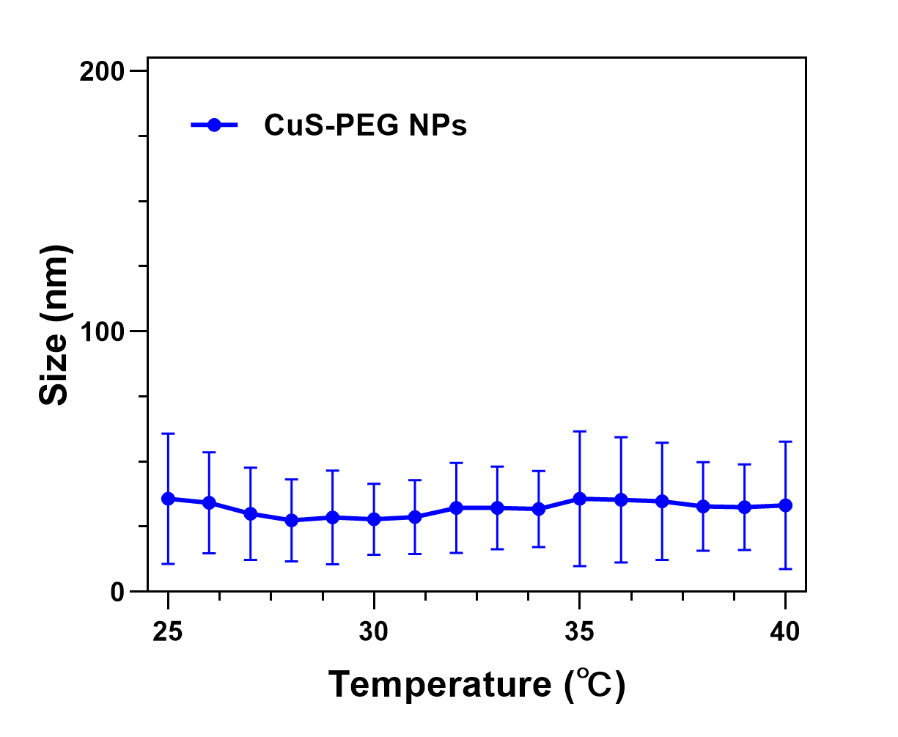


**Figure. S12.** The sizes of CuS-PEG NPs at 25~ 40 °C.


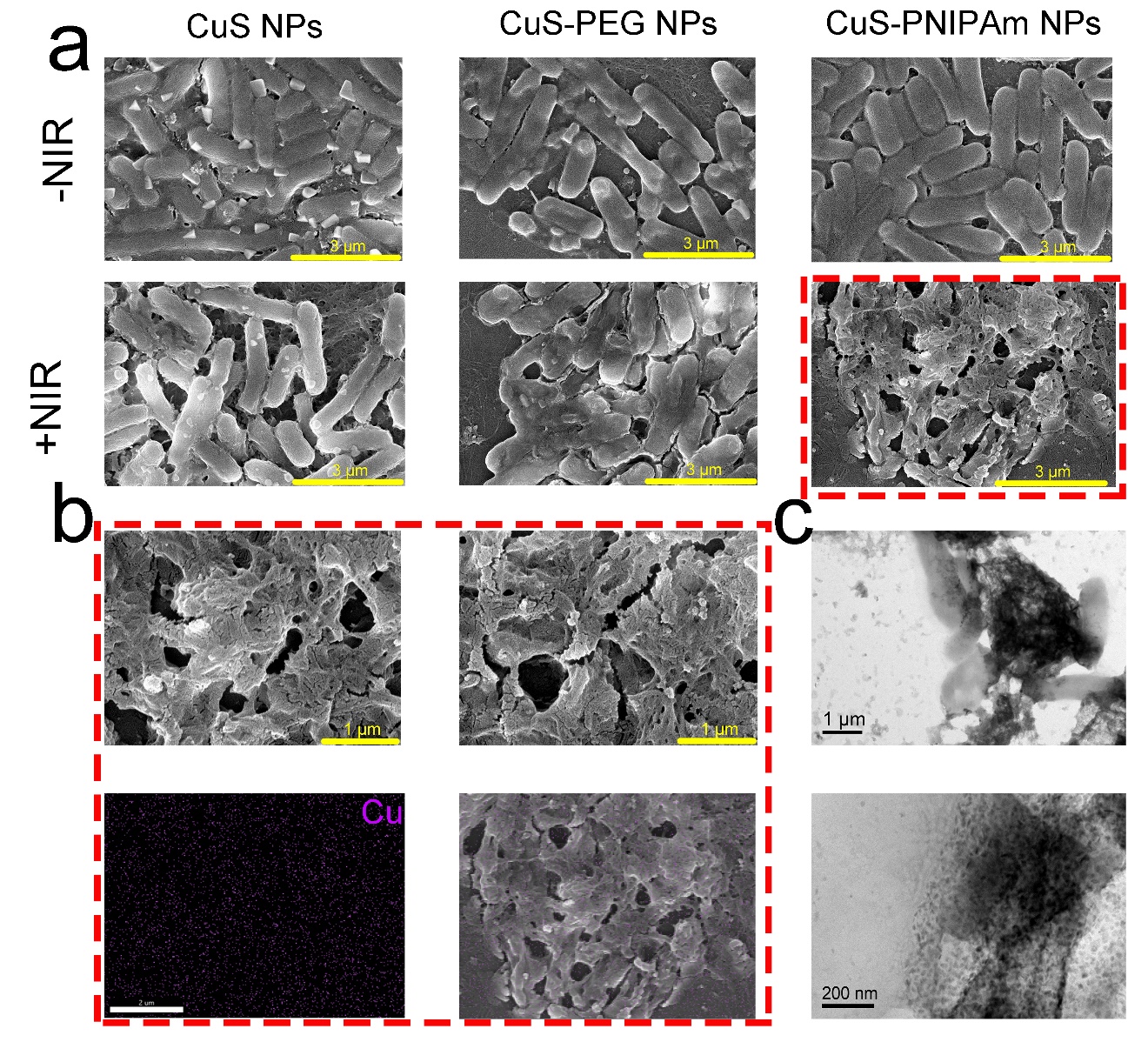


**Figure. S13.** Effect of NIR irradiation on bacteria in NPs solutions. (a) SEM images of E. coli in different solutions without or with NIR irradiation. (b) Local amplification of SEM image and EDAX mapping images of copper ions for E. coli in CuS-PNIPAm NPs irradiated with NIR. (c) TEM images of the aggregates of nanoparticles-nanoparticles and nanoparticles-bacteria induced by NIR irradiation. NIR irradiation proceeded for 5 min by a laser with 808 nm, 2 W/cm^2^.


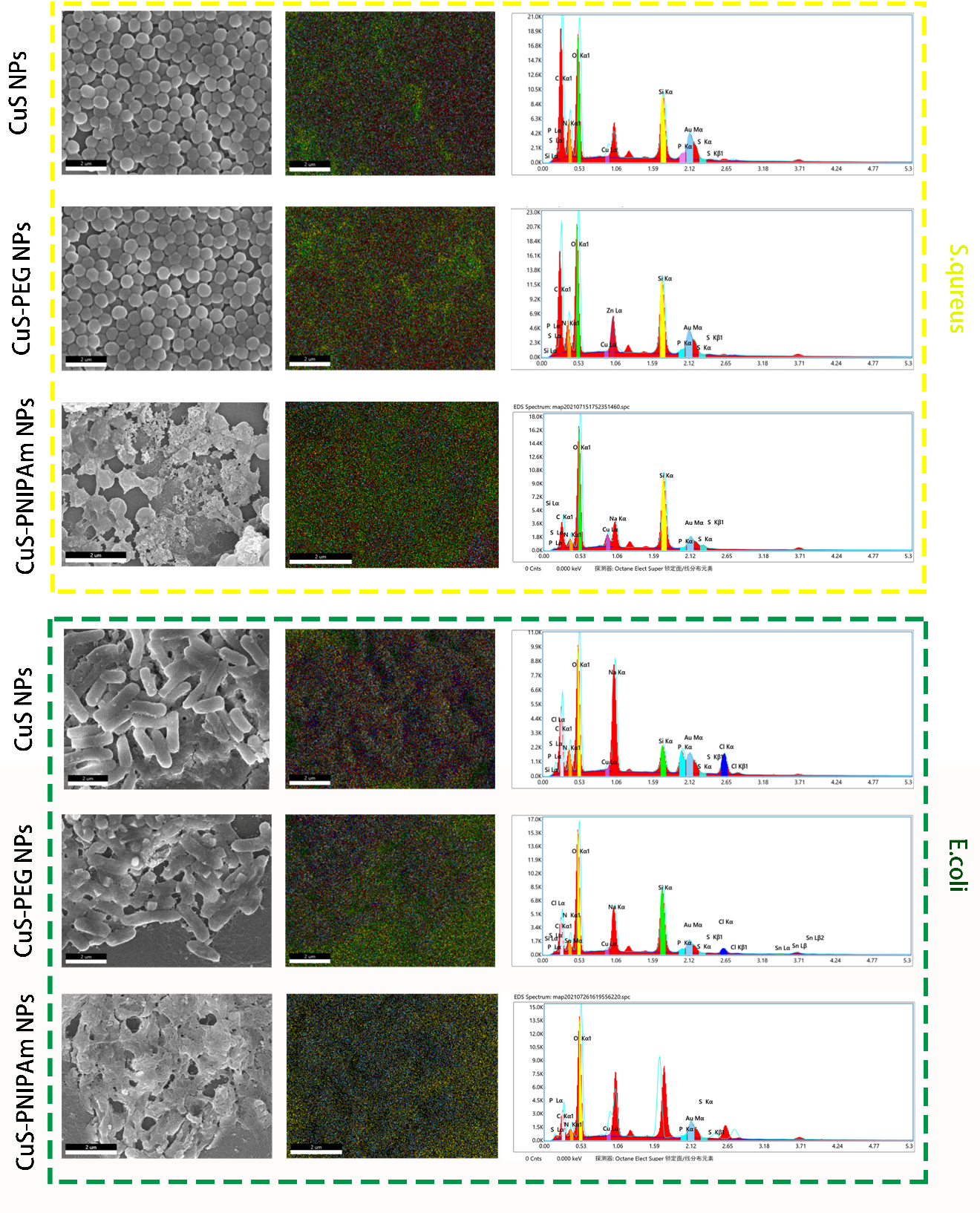


**Figure. S14.** EDAX mapping images for CuS NPs, CuS-PEG NPs and CuS-PNIPAm NPs mixed with S.aureus and E.coli.


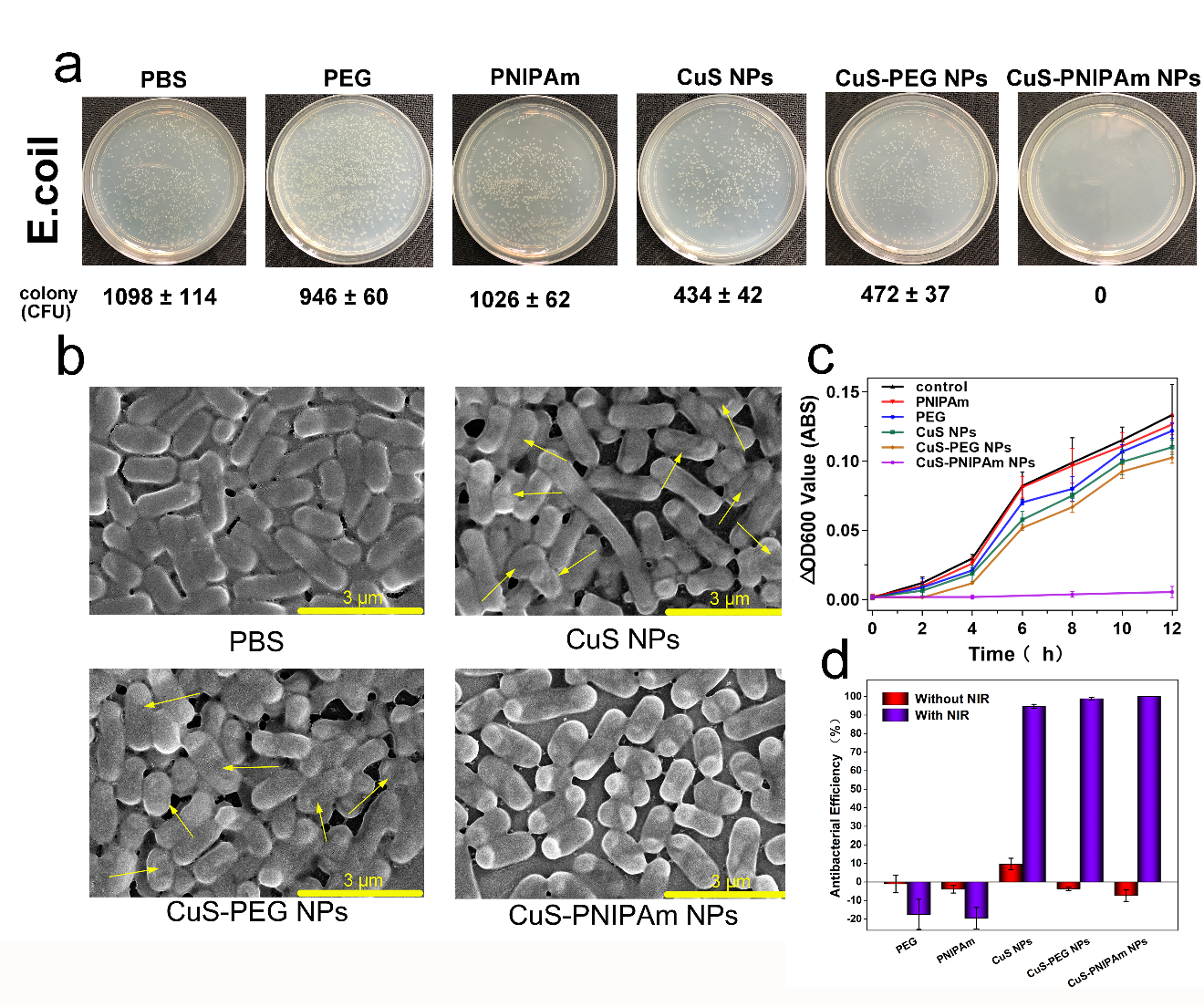


**Figure. S15.** Antibacterial effect of PTT in vitro. (a) Photographs of the LB ager plates, (b) SEM images and (c) OD 600 values for the E. coli cocultured with NPs solutions with NIR irradiation. (d) Antibacterial efficiencies against E. coli with and without NIR irradiation. The concentration for different solutions is 0.08 mM. NIR irradiation proceeded for 5 min by a laser with 808 nm, 2 W/cm^2^.


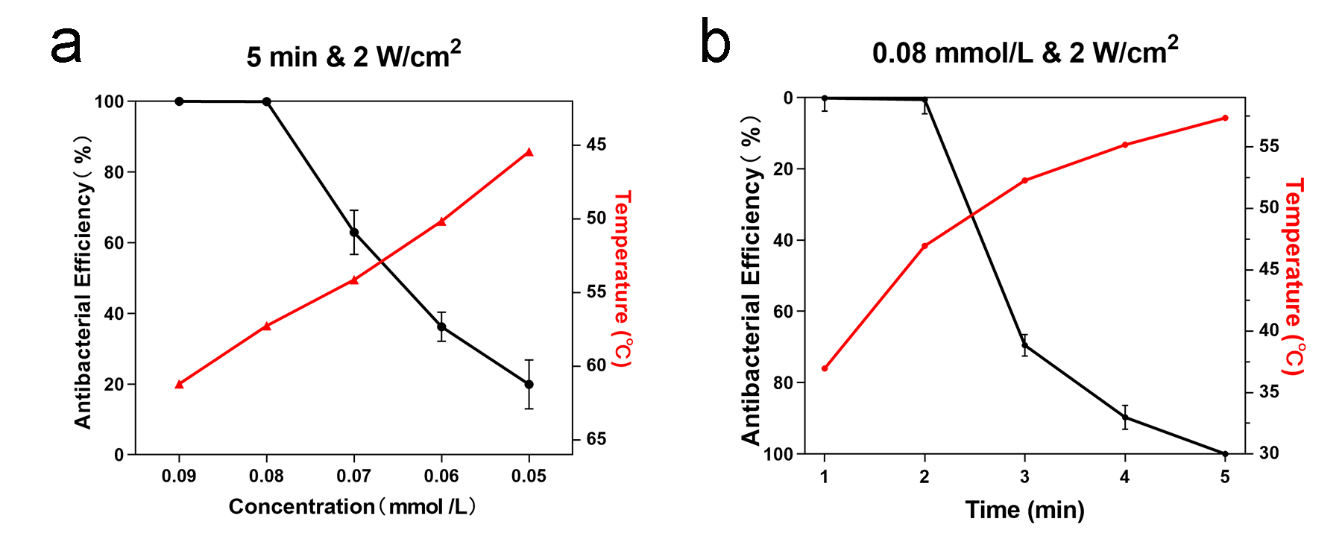


**Figure. S16.** The influences of CuS-PNIPAm NPs concentration (left) and irradiation time (right) on antibacterial efficiency against S. aureus.


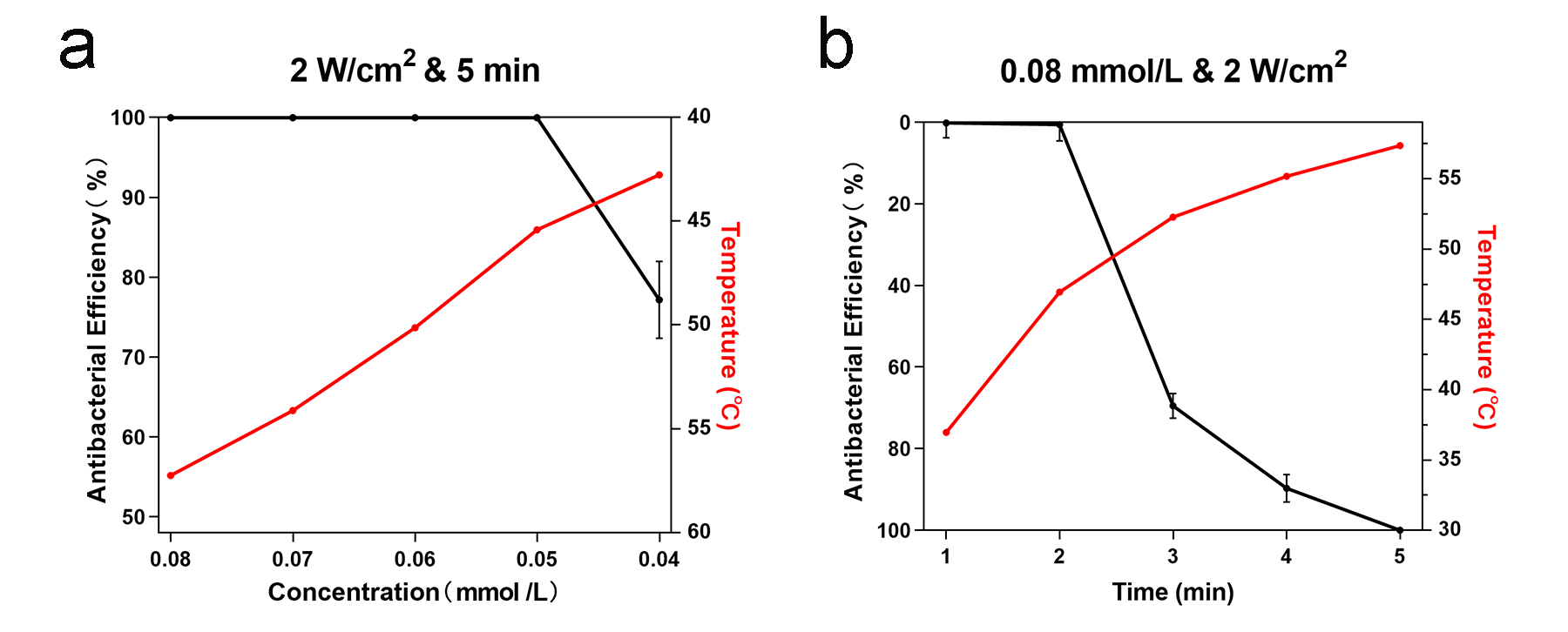


**Figure. S17.** The influences of irradiation time (left) and CuS-PNIPAm NPs concentration on the antibacterial efficiency against E. coli.


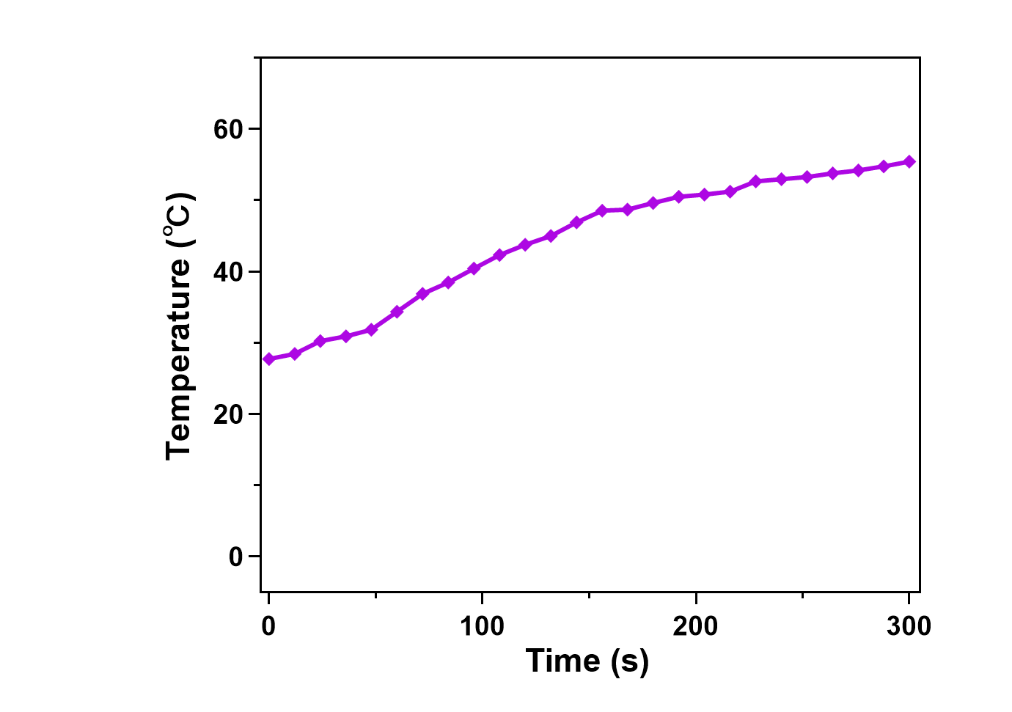


**Figure. S18.** Temperature evolution profiles for the mouse skin with CuS-PNIPAm NPs treament under NIR irradiation (0.2 mM, 2 W/cm^2^).
